# CD154 Induces Interleukin-6 Secretion by Kidney Tubular Epithelial Cells under Hypoxic Conditions: Inhibition by Chloroquine

**DOI:** 10.1155/2020/6357046

**Published:** 2020-01-31

**Authors:** Antoine Dewitte, Julien Villeneuve, Sébastien Lepreux, Marion Bouchecareilh, Xavier Gauthereau, Claire Rigothier, Christian Combe, Alexandre Ouattara, Jean Ripoche

**Affiliations:** ^1^Department of Anesthesia and Critical Care, CHU de Bordeaux, F-33600 Pessac, France; ^2^INSERM, UMR1026 Bioingénierie Tissulaire (Biotis), Université de Bordeaux, F-33000 Bordeaux, France; ^3^Department of Medical Genetics, Cambridge Institute for Medical Research, University of Cambridge, The Keith Peters Building, Cambridge Biomedical Campus, Cambridge CB2 0XY, UK; ^4^Pathology Unit, CH de Libourne, F-33505 Libourne, France; ^5^INSERM, UMR1053 Bordeaux Research in Translational Oncology (BaRITOn), Université de Bordeaux, F-33000 Bordeaux, France; ^6^CNRS, UMR5164, Université de Bordeaux, F-33000 Bordeaux, France; ^7^Department of Nephrology-Transplantation-Dialysis, CHU de Bordeaux, F-33076 Bordeaux, France; ^8^INSERM, UMR1034 Biology of Cardiovascular Diseases, Université de Bordeaux, F-33600 Pessac, France

## Abstract

Inflammation is a major contributor to tubular epithelium injury in kidney disorders, and the involvement of blood platelets in driving inflammation is increasingly stressed. CD154, the ligand of CD40, is one of the mediators supporting platelet proinflammatory properties. Although hypoxia is an essential constituent of the inflammatory reaction, if and how platelets and CD154 regulate inflammation in hypoxic conditions remain unclear. Here, we studied the control by CD154 of the proinflammatory cytokine interleukin- (IL-) 6 secretion in short-term oxygen (O_2_) deprivation conditions, using the HK-2 cell line as a kidney tubular epithelial cell (TEC) model. IL-6 secretion was markedly stimulated by CD154 after 1 to 3 hours of hypoxic stress. Both intracellular IL-6 expression and secretion were stimulated by CD154 and associated with a strong upregulation of IL-6 mRNA and increased transcription. Searching for inhibitors of CD154-mediated IL-6 production by HK-2 cells in hypoxic conditions, we observed that chloroquine, a drug that has been repurposed as an anti-inflammatory agent, alleviated this induction. Therefore, CD154 is a potent early stimulus for IL-6 secretion by TECs in O_2_ deprivation conditions, a mechanism likely to take part in the deleterious inflammatory consequences of platelet activation in kidney tubular injury. The inhibition of CD154-induced IL-6 production by chloroquine suggests the potential usefulness of this drug as a therapeutic adjunct in conditions associated with acute kidney injury.

## 1. Introduction

Accumulating evidence underscores the association and interdependence of hypoxic and inflammatory pathways. Indeed, hypoxia is a common feature of inflamed tissues, being linked for a large part to an unbalanced oxygen (O_2_) demand/supply [1]. Over the past years, the role of hypoxia in controlling inflammation has been increasingly appreciated [2]. Hypoxia is linked to the progression of inflammation via intricate mechanisms. On the one hand, hypoxia can stimulate the expression of proinflammatory cytokines via various pathways, including those involving the Hypoxia-Inducible Factor-1 (HIF-1) pathway; on the other hand, hypoxia also drives anti-inflammatory responses [2]. Hypoxia is also associated with an endoplasmic reticulum (ER) stress [3, 4], and the production of inflammatory cytokine secretion is an outcome of the ER stress [3, 4]. Nevertheless, much remains to be understood on how hypoxia pathways cooperatively operate on the different stages of inflammation. Hypoxia pathways not only drive pro- and anti-inflammatory responses but are also regulated, for example, by inflammatory mediators themselves, indicating complex feedback loops in the natural history of inflammation.

Acute kidney injury (AKI) is an important example of how inflammatory and hypoxic pathways interdepend. In sepsis and ischemia/reperfusion-associated AKI, current pathophysiological concepts give a significant role to tubulointerstitial inflammatory events [5]. Inflammation orchestrates the deleterious sequence of events that result in microcirculatory alterations and tubular cell injury in AKI and hypoxia is both a driver of injury and long-term outcome in AKI [6–8].

Among the plethora of inflammatory mediators involved, clinical and experimental studies have underscored the contribution of interleukin- (IL-) 6 to renal injury in kidney inflammatory conditions, including AKI [9]. IL-6 mediates acute inflammation in response to tissue damage, being involved in both inflammation promotion and resolution and tissue restorative response [10, 11], and has been implicated in inflammatory disorders [12]. Via the production of inflammatory mediators such as IL-6, tubular epithelial cells (TECs) contribute to AKI-associated inflammation [13]. Indeed, TECs are not only targets of inflammatory mediators but also active participants in kidney inflammation in response to inflammatory challenges, providing ground for self-amplifying inflammatory loops [14, 15]. The mechanisms of IL-6 production in a hypoxic/inflammatory context remain however ill understood. Additionally, constitutive and induced IL-6 expressions in TECs are not inhibited by glucocorticoids, emphasizing the need to understand how this cytokine is regulated for the management of kidney tubular inflammation [16].

Several studies support a role for platelets in driving tubular inflammation in AKI [17]. Hemodynamic disturbances and various inflammatory and coagulation pathways activate platelets resulting in the generation of a range of proinflammatory mediators. Among them, CD154, the ligand of CD40, is an upstream regulator of inflammation; the induction of proinflammatory cytokines is a major outcome of CD154 binding to CD40, as shown in various cells and TECs in particular [13, 18, 19]. CD154 is deleterious in AKI associated with ischemia/reperfusion, as the inhibition of CD40 signaling is protective, but underlying mechanisms remain unclear [20]. CD154 may be important in initiating and sustaining inflammation in kidney tubules via the induction of proinflammatory cytokines. However, little is known on how CD154 exerts its proinflammatory role in the context of hypoxia. Coincidental occurrence of inflammation and hypoxia and cross-regulations between hypoxic and inflammatory pathways underline the importance to understand how proinflammatory cytokine expression is regulated under hypoxic conditions. Here, we used HK-2 cells as a TEC model to study the control of IL-6 secretion by CD154 in O_2_ deprivation conditions. Looking for inhibitors of IL-6 induction by CD154, we examined the potential role of chloroquine, a drug long used in the treatment or prevention of malaria, which has found its application expanded to treat inflammatory diseases [21, 22].

## 2. Materials and Methods

### 2.1. Cell Culture

The immortalized human proximal TEC cell line HK-2 was from the American Tissue Type Culture Collection (LGC standards, Molsheim, France). Cultures were free of mycoplasma as evaluated by polymerase chain reaction (PCR) [23]. Cells were routinely grown in complete medium consisting of Dulbecco's modified Eagle's medium (DMEM, Gibco, ThermoFisher Scientific, Illkirch, France, glucose concentration 1 g/L), containing 10% fetal calf serum (FCS), 100 IU/mL penicillin, and 100 *μ*g/mL streptomycin. Cultures were routinely carried out under normoxic conditions. Experiments in hypoxic conditions (0.1% O_2_) were performed in a hypoxia incubator chamber (BioSpherix®, NY, USA). Culture media were deoxygenated for 24-48 hours at 0.1% O_2_ before use. Cells were grown in multiple well plates for various times. In some experiments, cultures were also performed in gas-permeable cell culture plates (Sarstedt, Marnay, France). For CD154 treatment, recombinant soluble CD154 (rsCD154, MegaCD40LTM, Coger SAS, Paris, France) was added at the beginning of hypoxic cultures and used routinely at a concentration of 100 ng/mL, a dose comparable to what was used in previous studies [24, 25]. In dose-effect experiments, rsCD154 was used at concentrations ranging from 1 to 200 ng/mL. Cytokines interleukin-1*α* (IL-1*α*), tumor necrosis factor-*α* (TNF-*α*), and interferon-*γ* (IFN-*γ*) were from ImmunoTools (Friesoythe, FRG). Cytokine concentrations (IL-1*α* (200 IU/mL), TNF-*α* (10 ng/mL, 200 IU/mL), or interferon-*γ* (200 IU/mL)) were chosen according to previous studies on CD40 expression [26, 27]. Hydrocortisone and acetylsalicylic acid (gift from Bordeaux Hospital) were used at 1 *μ*g/mL for hydrocortisone [28] and 50 *μ*g/mL for acetylsalicylic acid [29, 30], respectively. In actinomycin D experiments, actinomycin D (Sigma-Aldrich, Saint-Quentin-Fallavier, France) was used at a concentration of 5 *μ*g/mL and added to cells 30 mins before starting the culture in hypoxia. In cycloheximide experiments, cycloheximide (Sigma-Aldrich) was used at a concentration of 50 *μ*g/mL and added to cells 15 mins before starting the culture in hypoxia. In chloroquine experiments, cells were preincubated for 30 mins with chloroquine diphosphate salt (Sigma-Aldrich) at concentrations ranging from 12.5 to 100 *μ*g/mL before starting the culture in hypoxia.

### 2.2. Enzyme-Linked Immunosorbent Assay (ELISA)

Interleukin-6 (IL-6) concentrations in culture supernatants or in cell lysates were measured with commercial ELISA kits (R&D Systems, France, and RayBiotech, Tebu-Bio, France, respectively), according to the manufacturer's recommendations. For cell lysate measurements, cells were lysed in RIPA buffer (Sigma-Aldrich) supplemented with a protease inhibitor cocktail (Sigma-Aldrich). Cell lysates were prepared as described in Western Blotting. VEGF, IL-1*β*, and TNF-*α* in cell culture supernatants were measured with ELISA kits from R&D Systems.

### 2.3. PCR and Real-Time Quantitative PCR (RT-qPCR)

Total RNA was extracted from HK-2 cells using a RNA extraction kit (Macherey-Nagel, Hoerdt, France) following the manufacturer's instructions. RNA integrity was assayed using the Agilent RNA Screen Tape Assay Bioanalyzer 2200 (Agilent Technologies, Les Ulis, France). Complementary DNA (cDNA) was synthesized using hexaprimers and oligo-dT from 1 *μ*g of total RNA in a final volume of 20 *μ*L, using the First-Strand cDNA Synthesis Kit for RT-PCR (Quantitect Reverse Transcription Kit (Sigma-Aldrich)), according to the manufacturer's instructions. The qPCR was performed in duplicate on a CFX 384 (Bio-Rad, Marnes-la-Coquette, France) using SYBR® Premix Ex Taq™ bulk (Takara, Ozyme). Cycling parameters for the qPCR reaction included a 3 mins hot start followed by 40 cycles of denaturation at 90°C for 10 seconds, annealing at 60°C for 30 seconds, and elongation at 72°C for 30 seconds. Post-PCR melt curve was performed (65°C to 95°C/0.5° each 5 seconds). Ribosomal protein lateral stalk subunit P0 (*RPLP0*) and 18S rRNA were used as internal controls. Relative expression levels were calculated using the 2^−*ΔΔ*Ct^ method after normalization to the expression of the endogenous 18S rRNA reference gene and were presented as the fold increase relative to the control [31]. IL-6 primers were from Sino Biological (Interchim, Montluçon, France). CD40 and XBP1 primers used and measurement of the ratio spliced to unspliced *XBP1* mRNA (*XBP1*s/u) were performed as described [32].

### 2.4. Nascent RNA Experiments

Nascent RNAs were purified from total RNA using a Click-iT Nascent RNA Capture Kit (ThermoFisher Scientific) according to the manufacturer's instructions. Briefly, biotin-azide was attached to nascent 5-ethynyl uridine- (EU-) labelled RNA using click-it chemistry. The EU-labelled nascent RNAs were then purified using MyOne Streptavidin T1 magnetic Dynabeads (ThermoFisher Scientific). cDNA synthesis was then performed on magnetic bead-captured RNAs, using the VILO cDNA synthesis kit (ThermoFisher Scientific) and real-time qPCR performed. RNA final concentration and purity (OD 260/280) were determined using a NanoPhotometer® P 330 (Implen GmbH, Munich, Germany). Real-time qPCR experiments were performed in duplicate using a Takyon ROX SYBR 2X MasterMix dTTP blue (Kaneka Eurogentec S.A., Belgium) using cDNA diluted 1 : 50 and the following program: 95°C for 5 min as initial denaturation and 40 cycles of 95°C for 15 sec and 61°C for 30 sec for amplification in a CFX Connect™ Real-Time PCR Detection System (Bio-Rad Laboratories, Hercules, CA, USA). Target gene expression was quantified using the cycle threshold (Ct) values, and relative expression levels were calculated according to the 2^−*ΔΔ*Ct^ method using *RPLP0* as reference gene. Primers used for IL-6 were from Sino Biological, and primer sequences used for *RPLP0* were 5′ CCTCGTGGAAGTGACATCGT 3′ and 5′ ATCTGCTTGGAGCCCACATT 3′.

### 2.5. Western Blotting

Cells were lysed in RIPA buffer (Sigma-Aldrich) supplemented with a protease inhibitor cocktail (Sigma-Aldrich). Cell lysate were obtained after centrifugation at 15000g for 10 mins, at 4°C. Protein quantification was performed using the Pierce BCA Protein Assay kit (ThermoFisher Scientific). Samples were denatured in SDS-PAGE buffer containing 2% SDS and 5% *β*-mercaptoethanol. Samples, 10 *μ*g protein, were then separated on a 10 or 15% acrylamide-bis acrylamide gel. After transfer (Immobilon-P membrane, Merck Millipore, Molsheim, France), membranes were blocked with TBST (TBS containing 0.1% Tween 20) containing 2.5% bovine serum albumin (BSA) and then incubated overnight with the indicated antibody at 1 *μ*g/mL at 4°C. After several washes with TBST, the membrane was incubated for 1 hour at RT with the appropriate secondary antibody conjugated to HRP. The detection was performed using an enhanced chemiluminescent substrate, ECL plus (GE Healthcare, Aulnay-sous-Bois, France), and images were analyzed with an Image Quant LAS 4000 mini camera.

### 2.6. Immunofluorescence Microscopy

Cells grown on coverslips were fixed in 4% paraformaldehyde (PFA) for 10 mins. For permeabilization, after several washes with TBS, cells were incubated for 10 mins in 0.1% Triton X100, washed in TBS, and incubated in blocking buffer (TBS containing 1% BSA and 1% FCS). Cells were then incubated with a mouse monoclonal anti-CD40 antibody, at a 1/100 dilution (Santa-Cruz Biotechnology, Heidelberg, FRG) in blocking buffer, for 2 hours, at RT. After several washes, cells were incubated with Alexa Fluor® 488 goat anti-mouse antibody (Molecular Probes, Invitrogen, France) at a 1/200 dilution, for 1 hour at RT. After several washes, nuclei were labelled with 1 *μ*g/mL of DAPI solution for 1 min. Then, coverslips were mounted on slides using Fluoromount-G (SouthernBiotech, USA). For negative control, cells were incubated with matched isotype immunoglobulin and secondary antibody. Samples were analyzed with a confocal microscope (Leica Microsystems, France) and Leica software.

### 2.7. Flow Cytometry

Cytometry was performed on an Epics XL2 (Beckman Coulter) flow cytometer working under the EXPO 32 ADC software (Beckman Coulter). HK-2 cells were incubated for 2 hours at RT with anti-CD40 monoclonal antibodies (Santa-Cruz Biotechnologies, clone H-10, Clinisciences, France) at 10 *μ*g/mL in phosphate-buffered saline (PBS) containing 1% BSA. Cells were washed once with PBS and further incubated for 30 mins at RT with a 1/400 dilution of Alexa Fluor 488 (Molecular Probes) secondary antibody.

### 2.8. Viability, Apoptosis, Toxicity, and Proliferation Assays

Lactate dehydrogenase Pierce colorimetric kit assay (Life Technologies SAS, Courtaboeuf, France) was used to measure cellular cytotoxicity. Cell counts were performed on a Malassez cell. Anti-human Fas agonistic mAb 7C11 antibody, a gift of F Belloc, Université de Bordeaux, was used in apoptosis experiments. Apoptosis measurement was performed by flow cytometry with the annexin V-FITC apoptosis staining/detection kit (Abcam, France).

### 2.9. Antibodies Used in This Study Are Described in Supplemental Material and Method Section

#### 2.9.1. Statistical Analysis

Data were generated from independent experiments and are presented as mean ± SD. Comparison between groups was determined using the one-sample *t*-test, Mann-Whitney test, or Kruskal-Wallis test as appropriate. All tests were two-sided, and a *p* value < 0.05 was taken to indicate statistical significance. Statistical analyses were performed using GraphPad Prism version 6.00 (GraphPad Software, La Jolla, CA, USA).

## 3. Results

### 3.1. HK-2 Cells Are a Relevant Cellular Model to Assess CD154-CD40 Function

**Figure 1:**
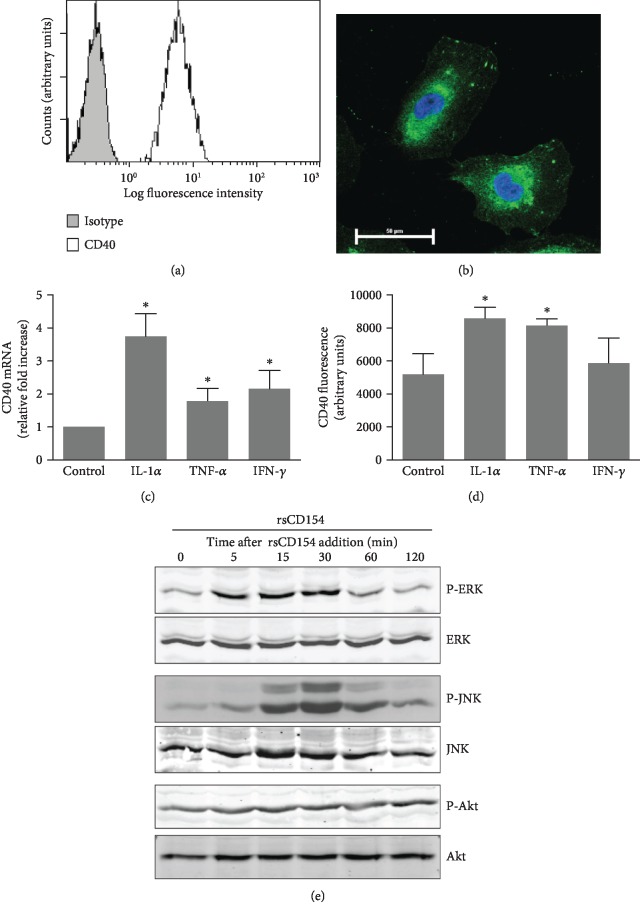
CD40 expression by HK-2 cells. (a, b) CD40 expression by HK-2 cells was analyzed by flow cytometry to assess the surface expression of CD40 (a) and immunofluorescence microscopy to assess the total expression (surface and intracellular pool) (b; nuclei are counterstained with DAPI). (c, d) HK-2 cells were treated with IL-1*α* (200 IU/mL), TNF-*α* (200 IU/mL), or interferon-*γ* (200 IU/mL) for 24 hours and CD40 mRNA expression analyzed by RT-qPCR (c) (*n* = 4, ^∗^significant relatively to control), or by flow cytometry (d) (*n* = 3, ^∗^significant relatively to control). (e) Treatment of HK-2 cells with rsCD154 activates ERK and JNK pathways. HK-2 cells were incubated with rsCD154 and cells lysed at indicated time points. Cell lysates were subjected to SDS-PAGE and immunoblotted with indicated antibodies.

**Figure 2:**
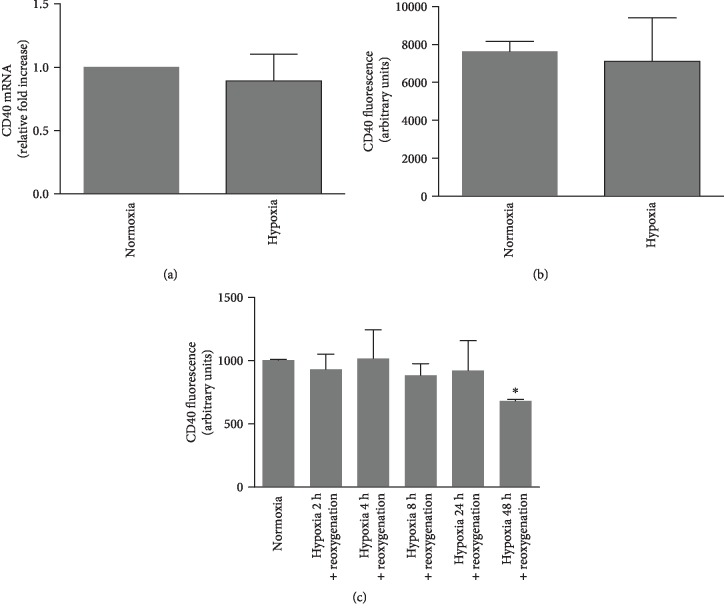
CD40 expression is not modified by hypoxic stress. (a, b) HK-2 cells were incubated under normoxic or hypoxic conditions during 24 hours and CD40 expression analyzed by RT-qPCR (a) (*n* = 3) or flow cytometry (b) (*n* = 3). (c) HK-2 cells were subjected to different hypoxic times followed by a 24-hour culture in normoxic conditions and CD40 expression analyzed by flow cytometry (*n* = 3, ^∗^significant relatively to normoxic control).

In order to investigate the control of IL-6 secretion by CD154 on HK-2 cells, we first assessed the expression and functionality of the CD40, the main receptor of CD154, which localizes at the cell surface after its transport along the secretory pathway. As expected [33], HK-2 cells expressed CD40 at the cell surface as monitored by flow cytometry (Figure 1(a)) and its total expression (surface and intracellular pool) was confirmed by immunofluorescence microscopy (Figure 1(b)). As previously reported [18], CD40 mRNA expression was stimulated by inflammatory mediators such as TNF-*α*, IFN-*γ*, and IL-1*α* (Figure 1(c)). IL-1*α* was a strong inducer of CD40 at both mRNA and protein levels [14] (Figures 1(c) and 1(d)). CD154 binding to CD40 on HK-2 cells was functional as shown by the corresponding activation of known downstream effectors, extracellular signal-regulated (ERK) and c-Jun N-terminal (JNK) kinases (Figure 1(e)) [34]. We next studied whether CD40 expression was modified by hypoxic conditions. CD40 mRNA and protein expression were not modified by hypoxic culture conditions or following a reoxygenation step up to 24 hours hypoxic challenge (Figures 2(a)–2(c)). A moderate but significant reduction of CD40 expression was observed following a 48-hour hypoxic period (Figure 2(c)), which could be related to various mechanisms, including reduced transcription and protein synthesis, that we have not explored further.

### 3.2. Resistance of HK-2 Cells to Hypoxic Conditions and Association to Stress Responses

**Figure 3:**
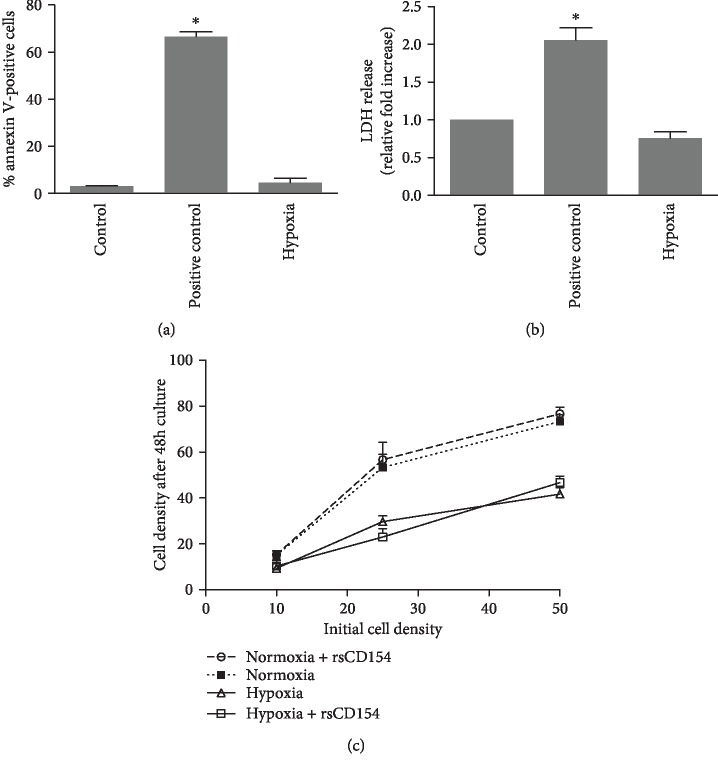
HK-2 cells are tolerant to hypoxic challenge. HK-2 cells were subjected to a hypoxic stress for 24 hours and cell viability assayed by measuring annexin-positive cells (a) (*n* = 3, ^∗^significant relatively to control) (positive control: cells treated with anti-Fas antibody 1 *μ*g/mL) or LDH release assay (b) (*n* = 4, ^∗^significant relatively to control) (positive control from the LDH cytotoxicity assay kit). (c) Hypoxia mitigates HK-2 cell growth. HK-2 cells were plated at various densities, cultures performed under hypoxic or normoxic conditions and cells counted.

We next studied the functional responses of HK-2 cells to hypoxic challenge by incubating cells in O_2_ deprivation conditions. Previous studies already demonstrated that 1% O_2_ conditions for up to 48 hours of culture did not induce apoptosis or necrosis in HK-2 cells [35, 36]. We analyzed HK-2 cell susceptibility to more severe hypoxic stress, 0.1% O_2_ conditions during 24 hours. As reported for 1% O_2_ culture conditions, no significant increase of apoptosis or loss of viability following hypoxic challenge (Figures 3(a) and 3(b)) or following hypoxia/reoxygenation (not shown) was detectable. O_2_ deprivation mitigated cell growth, and rsCD154 did not stimulate HK-2 proliferation in normal growth condition or hypoxic condition (Figure 3(c)).

**Figure 4:**
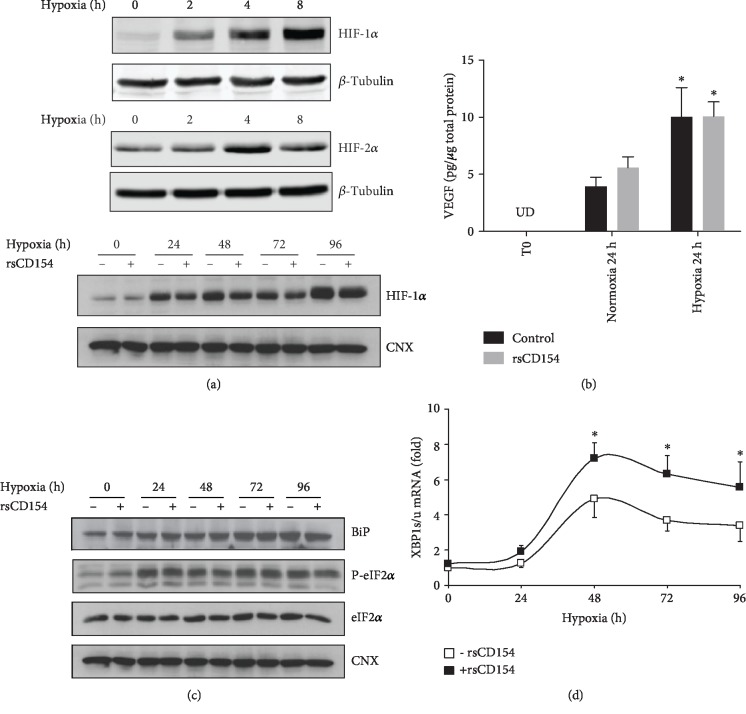
Induction of HIF-1*α* and HIF-2*α* and endoplasmic reticulum stress in HK-2 cells grown under hypoxic conditions; (a) HIF-1*α* and HIF-2*α* are induced in response to hypoxic stress. Top panel: HK-2 cells were grown under hypoxic conditions for the indicated times and immunoblotting with anti-HIF-1*α* or anti-HIF-2*α* antibodies performed on cell lysates (*β*-tubulin was used as loading controls); bottom panels: cells were grown under hypoxic conditions for the indicated times in the presence or not of 100 ng/mL rsCD154 and immunoblotting with anti-HIF-1*α* antibody performed on cell lysates (calnexin (CNX) was used as loading controls). (b) VEGF concentration increases in HK-2 cell supernatants in response to hypoxic stress. VEGF was quantified by ELISA in supernatants of HK-2 cells grown under hypoxic or normoxic conditions (*n* = 3, ^∗^significant relatively to normoxic control; UD: undetectable levels). (c) Hypoxia induces time-dependent endoplasmic reticulum stress features in HK-2 cells. HK-2 cells were grown under hypoxic conditions for the indicated times and immunoblotting with antibodies against BiP, eIF2*α*, and Phospho-eIF2*α* (P-eIF2*α*) performed on cell lysates (calnexin (CNX) was used as a loading control). (d) Fold induction of the spliced/unspliced ratio of *XBP-1* (*XBP1* s*/u*) mRNA in HK-2 cells grown under hypoxic conditions for the indicated times in the presence or absence of rsCD154 (*n* = 3, ^∗^significant relatively to time 0).

We next examined the activation of several stress pathways induced under hypoxic condition. First, we assessed in the experimental conditions used the expression of two transcription factors, HIF-1*α* and HIF-2*α*, which are upregulated in hypoxic conditions and whose activity controls the expression of several genes essential for cell adaption under hypoxic stress. As expected, O_2_ deprivation increased HIF-1*α* and HIF-2*α* protein expression in HK-2 cells (Figure 4(a)); treatment of HK-2 cells with rsCD154 did not modify HIF-1*α* expression (Figure 4(a)). We also studied the expression of one HIF-1*α* target genes, vascular endothelial growth factor (VEGF), and observed that 0.1% O_2_ hypoxic condition led to the accumulation of VEGF in culture supernatants (Figure 4(b)). In these conditions, treatment of cells with rsCD154 did not affect VEGF expression (Figure 4(b)).

Second, we assessed whether hypoxic conditions induced ER stress and its adaptive response, the Unfolded Protein Response (UPR), which are commonly associated with hypoxia [37]. To study ER stress, we monitored UPR markers, such as BiP/GRP78, phosphorylated eukaryotic translation-initiation factor 2*α* (eIF2*α*), and the expression of the alternative spliced form of *XBP-1* mRNA. ER stress induction depended on the duration of hypoxia. Indeed, ER stress markers were not detectable for short hypoxic challenges such as 1 or 3 hours and could only be detected at 24 hours and later times (Figure 4(c)). Increased splicing of *XBP-1* mRNA was also only observed at late times depicting bell-shaped curve kinetics (Figure 4(d)) and CD154 increased *XBP1* mRNA splicing (Figure 4(d)) as previously reported [32]. In a condition mimicking anoxia in which cells were incubated for 1 hour with 50 ng/mL antimycin A, a mitochondrial respiratory chain blocking agent, *XBP-1* mRNA splicing was only minimally induced and no effect of CD154 was detectable. However, upon the removal of antimycin A, mimicking reoxygenation, *XBP-1* mRNA splicing was further increased and the enhancing effect of CD154 was apparent (Supplemental [Supplementary-material supplementary-material-1]).

Altogether, these results showed that HIF-1*α* and HIF-2*α* inductions are early features of hypoxic stress in HK-2 cells and that ER stress could only be observed at late culture times, being likely related to the addition of other ER stress-promoting signals during the progression of cell culture, such as glucose depletion [38]. No effects of rsCD154 on HIF-1*α* or on ER stress markers were observed at early hypoxia times.

### 3.3. CD154 Stimulates IL-6 Secretion by HK-2 Cells in Hypoxic Conditions

**Figure 5:**
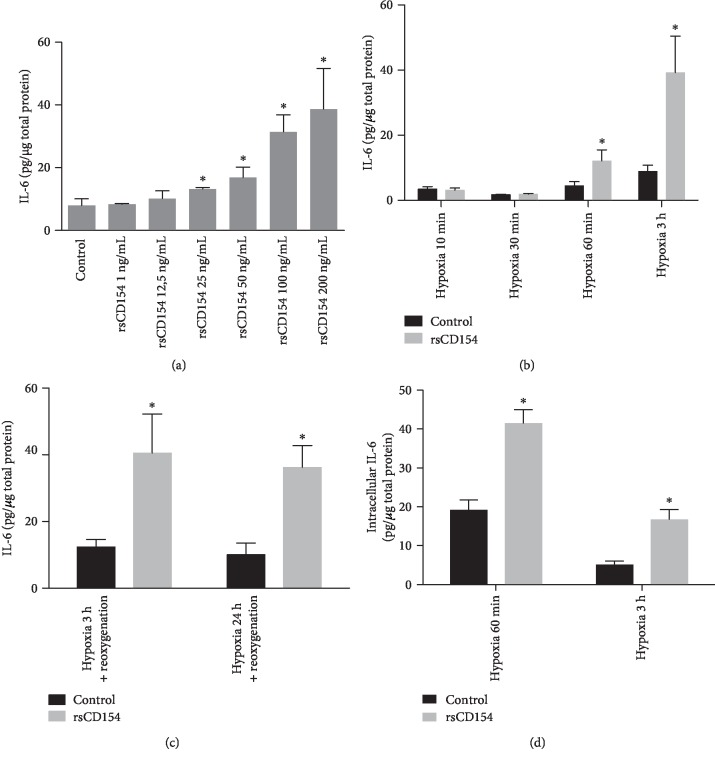
CD154 stimulates early IL-6 production by HK-2 cells under hypoxic conditions. (a) HK-2 cells were grown under hypoxic conditions for 3 hours in the presence or not of increasing rsCD154 concentrations and IL-6 measured by ELISA in cell culture supernatants (*n* = 3, ^∗^significant relatively to control). (b) HK-2 cells were grown under hypoxic conditions for the indicated times in the presence or not (control) of rsCD154 and IL-6 measured by ELISA in cell culture supernatants (*n* = 3, ^∗^significant relatively to control). (c) HK-2 cells were grown under hypoxic conditions for 3 or 24 hours, followed by a 24-hour culture in normoxic conditions in the presence or not (control) of rsCD154 and IL-6 measured by ELISA in cell culture supernatants (*n* = 4, ^∗^significant relatively to control). (d) HK-2 cells were grown 1 or 3 hours under hypoxic conditions in the presence or not (control) of rsCD154 (control) and IL-6 concentrations measured in cell lysates (*n* = 3; ^∗^significant relatively to control).

We then investigated the regulatory role of CD154 on IL-6 secretion in short-term hypoxic stress conditions, with the aim to study the contribution of CD154 in the regulation of the expression of inflammatory mediators in hypoxic conditions. We worked with short stimulation times to remain close to pathophysiological conditions encountered during acute inflammation. IL-6 was investigated as it is a primary mediator of acute inflammation, together with IL-1*β* and TNF-*α*, cytokines also involved at the early stages of inflammation [39]. To determine the rsCD154 concentration, dose-response effect experiments were performed. We observed that rsCD154 dose-dependently stimulated the expression of IL-6 after a 3-hour hypoxic stress with a net increase at 100 ng/mL and no significant difference between doses of 100 and 200 ng/mL (Figure 5(a)).

We next studied the regulatory role of rsCD154 on IL-6 secretion in various hypoxic stress short-term periods. IL-6 secretion was not induced by a short hypoxic stress of 10 and 30 min upon treatment with 100 ng/mL rsCD154 (Figure 5(b)). The secretion of IL-6 was upregulated in the presence of rsCD154 after a 1- or 3-hour hypoxic stress (Figure 5(b)) and after hypoxia-reoxygenation (Figure 5(c)). We also tested whether, in hypoxic conditions, rsCD154 regulated the expression of IL-1*β* and TNF-*α* cytokines. Neither IL-1*β* nor TNF-*α* was induced upon brief hypoxic challenge for 3 hours, even in the presence of rsCD154 at 100 ng/mL (data not shown). IL-6 measurements in cell lysates confirmed secretion studies, showing that, under hypoxic conditions, treatment with rsCD154 stimulated intracellular IL-6 content following a 1- or 3-hour hypoxic stress (Figure 5(d)). A reduced intracellular IL-6 expression level was observed after 3 hours alongside to the increased IL-6 secretion. Additional mechanisms may be triggered to prevent excessive upregulation of IL-6 expression upon O_2_ deprivation. The induction of IL-6 secretion by rsCD154 in hypoxia was not due to differences in cell death or proliferation (Figures 3(a)–(c)). A similar induction of IL-6 secretion was observed also in gas-permeable plates (Supplemental [Supplementary-material supplementary-material-1]). Altogether, the rapid CD154-mediated induction of IL-6 in hypoxic conditions suggested a role for CD154 in an early control of IL-6 production by HK-2 cells in conditions associated with hypoxia.

### 3.4. CD154 Is a Strong Inducer of IL-6 mRNA Expression in HK-2 Cells in Hypoxic Conditions

**Figure 6:**
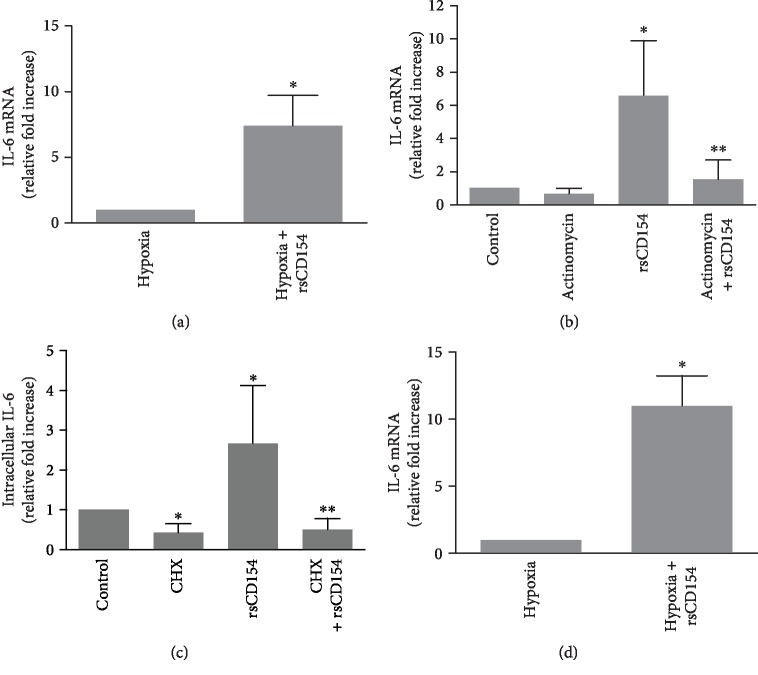
CD154 induces IL-6 mRNA in HK-2 cells grown under hypoxic conditions. (a) HK-2 cells were grown 3 hours under hypoxic conditions in the presence or absence of rsCD154 and IL-6 mRNA expression analyzed by RT-qPCR. Data are normalized to cells grown in hypoxic conditions in the absence of rsCD154 (*n* = 6; ^∗^significant relatively to control condition). (b) HK-2 cells were grown 3 hours under hypoxic conditions in DMEM containing or not rsCD154 and actinomycin D, and IL-6 mRNA expression analyzed by RT-qPCR. Data are normalized to cells grown in control condition (*n* = 6, ^∗^significant relatively to control, ^∗∗^significant relatively to rsCD154 condition). (c) HK-2 cells were grown 1 hour under hypoxic conditions in the presence or not of rsCD154 and cycloheximide (CHX), cells were lysed, and IL-6 was measured in cell lysates by ELISA (*n* = 4, total protein amount in cell lysates did not differ between conditions; ^∗^significant relatively to control, ^∗∗^significant relatively to rsCD154 condition). (d) CD154 stimulates *IL-6* gene transcription in hypoxic conditions. HK-2 cells were grown 3 hours under hypoxic conditions in the presence or absence of rsCD154 in DMEM, and a nascent RNA capture assay was used to monitor the change in *de novo* IL-6 mRNA synthesis. Data are normalized to cells grown in hypoxic condition without rsCD154 (*n* = 4, ^∗^significant relatively to control).

The addition of rsCD154 stimulated the expression of IL-6 mRNA in HK-2 cells in hypoxic conditions (Figure 6(a)). Therefore, the stimulation of IL-6 production by CD154 in hypoxic conditions was associated with an increased expression of IL-6 mRNA, and underlying mechanisms were next studied. rsCD154 treatment did not result in an increased IL-6 mRNA abundance when actinomycin D was added to inhibit transcription (Figure 6(b)). The addition of cycloheximide resulted in a decrease of IL-6 protein expression within one hour in HK-2 cells, indicating instability of the IL-6 protein; however, no stabilization of IL-6 protein by CD154 could be observed (Figure 6(c)). Altogether, these results indicated a rapid and strong induction of IL-6 mRNA expression by HK-2 cells in hypoxia. The absence of detectable mRNA increase by CD154 in experiments in which transcription was blocked by actinomycin D suggested regulation acting at the transcriptional level. To monitor the change in *de novo* IL-6 mRNA synthesis induced by CD154 in hypoxic conditions, we use a nascent RNA capture assay on HK-2 cells grown 3 hours under hypoxic conditions. Results (Figure 6(d)) showed that CD154 stimulates the transcription of *IL-6* gene under hypoxic conditions.

### 3.5. Chloroquine Alleviates the CD154-Mediated Induction of IL-6 in HK-2 Cells

**Figure 7:**
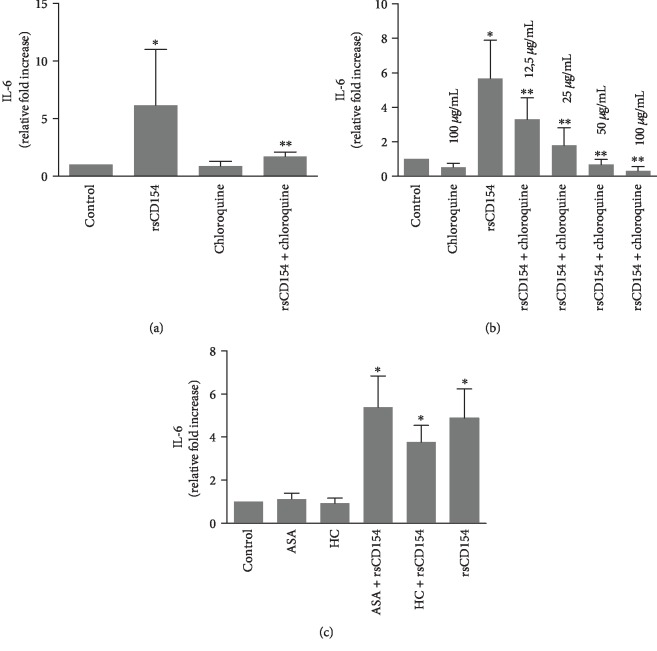
Chloroquine alleviates the CD154-mediated induction of IL-6 in HK-2 cells. (a) HK-2 cells were preincubated with chloroquine (50 *μ*g/mL) and then incubated for 3 hours at 0.1% O_2_ in the presence or not of rsCD154; IL-6 protein was measured by ELISA in the cell culture supernatants (*n* = 6, ^∗^significant relatively to control, ^∗∗^significant relatively to rsCD154 condition). (b) Dose effect experiments: HK-2 cells were preincubated with the indicated concentrations of chloroquine and then incubated for 3 hours at 0.1% O_2_ in the presence or not of rsCD154; IL-6 protein was measured by ELISA in the cell culture supernatants (*n* = 7, ^∗^significant relatively to control, ^∗∗^significant relatively to rsCD154 condition). (c) HK-2 cells were incubated for 3 hours at 0.1% O_2_ in the presence or not of rsCD154, hydrocortisone (HC) and acetylsalicylic acid (ASA), and IL-6 protein measured by ELISA in cell culture supernatants (*n* = 4, ^∗^significant relatively to control).

Discovering inhibitors of the induction of IL-6 in inflammatory conditions is an important medical issue; this is particularly true for AKI in sepsis systemic inflammatory response that remains a devastating complication due to the lack of efficient treatment. An efficient control of inflammation has not yet been attained in this context. Chloroquine is acknowledged to have anti-inflammatory properties, as for example, its administration result in lowering proinflammatory cytokines in patients with inflammatory conditions [22]. We therefore examined the potential role of chloroquine in the CD154-mediated induction of IL-6 secretion in HK-2 cells following a 3-hour hypoxic stress (Figure 7(a)). The induction of IL-6 secretion by CD154 was dose-dependently reduced in chloroquine-treated HK-2 cells (Figure 7(b)). Taken together, these results suggested that chloroquine is able to reduce the induction by CD154 of IL-6 secretion in HK-2 cells. We also tested steroidal and nonsteroidal anti-inflammatory drugs, such as hydrocortisone (HC) and acetylsalicylic acid (ASA) but did not observe a reduction of CD154-mediated induction of IL-6 secretion upon treatment with hydrocortisone or acetylsalicylic acid at the dose and conditions that were used (Figure 7(c)).

## 4. Discussion

Proinflammatory cytokines are key players in the progression of inflammation, and much remains to be understood on mechanisms that regulate the cytokine network in hypoxia. Results of our study indicate that CD154 is an early and strong inducer of IL-6 secretion in HK-2 cells grown under O_2_ deprivation conditions. The induction of IL-6 secretion by hypoxic stress *per se* remains debated; lack of or moderate inductions of IL-6 protein secretion by hypoxic stress have been described in other cellular models; a significant IL-6 induction may require prolonged hypoxia or reoxygenation steps. Hypoxic stress was found associated with an increased expression of IL-6 mRNA, as shown in fibroblasts, vascular smooth cells, endothelial cells, astrocytes or cardiac myocytes, and TECs, after a brief or a prolonged hypoxic challenge [40–44]. Mechanisms linking signaling pathways activated by hypoxia to the increased expression of IL-6 mRNA remain to be fully understood; however, some cues may be brought by transcription studies that show increased transcription of the *IL-6* gene upon hypoxic stress [40–43]. Indeed, the *IL-6* gene promoter contains a hypoxia-responsive element [45].

The stimulation of IL-6 secretion by CD154 suggests a regulatory role for CD40 signaling in IL-6 production under hypoxic conditions. Lack of stabilization of IL-6 protein or mRNA and measurement of nascent RNAs indicates that a main mechanism underlying CD154 stimulatory effect is stimulation of IL-6 transcription. CD40 signaling controls the transcription of *IL-6* gene in a variety of cells [46, 47]. IL-6 mRNA is unstable, which is the case for several cytokines and growth factors, a phenomenon believed to be critical to the kinetics of the inflammatory response; however, there is little characterization of IL-6 posttranscriptional regulators, and the regulation of *IL-6* gene expression may be to a large extent transcriptional. IL-6 was also found in intracellular stores in some cells, allowing another potential mechanism for rapid IL-6 mobilization [48, 49].

Rapid and sustained induction of IL-6 secretion is likely to be necessary to mount an immediate inflammatory response in the context of tissue injury. In an inflammatory environment, there are multiple potential sources for CD154, as various immune cells express CD154 and release its soluble form when stimulated by inflammatory signals [18]. Platelets are the principal reservoir of CD154 in blood, being readily present and activated in inflamed tissues an important mechanism that provides CD154 in the inflammatory milieu [50]. *In vivo*, higher platelet reactivity and response to agonists observed in low O_2_ conditions suggest that hypoxia favors platelet activation [51, 52]. Whether *in vivo* hypoxia *per se* is an inducer of platelet activation remains debated. It must be stressed, however, that the *in vivo* hypoxic/inflammatory environment is a complex one and can lead to platelet activation via multiple mechanisms, including the liberation of alarmins. Platelet activation in AKI is likely to result from gathered mechanisms, including blood flow impairment, ongoing inflammation, and hypoxia in the tubular microcirculation, leading to CD154 expression on platelets, the release of sCD154, and the release of CD154-expressing microvesicles. Indeed, there are signs of platelet activation in ischemic or sepsis AKI rodent models [53]. The rapid kinetics of CD154 expression make CD154 a potential front line mediator of inflammation in the tubular microenvironment. In the ischemic or sepsis-associated AKI, multiple inflammation modifiers concur to tubular epithelium injury, lesion, and regeneration [7]. Such a multiplicity underlines the difficulty of addressing the responsibility of a particular pathway. However, the powerful induction of IL-6 production by CD154 in hypoxia may be part of the CD154 proinflammatory deleterious effects. Indeed, the release of cytokines at site of injury is a hallmark feature of the early inflammatory response, and IL-6 is a key pathophysiological player in AKI tubular injury [54, 55]. Moreover, IL-6 serum level is an early marker of organ dysfunction and predicts poor outcome and renal recovery in critically ill patients with AKI [56]. Interestingly, the inhibition of CD40 signaling has demonstrated encouraging protective effects in a kidney ischemia-reperfusion injury model [20]. The initial steps of acute inflammation are dependent on early response cytokines such as IL-1*β*, TNF-*α*, and IL-6 [39]. However, we did not observe an upregulation of IL-1*β* and TNF-*α* even in the presence of CD154 within a 3-hour short-term hypoxic stress (data not shown). Although we have not studied other proinflammatory cytokines, it is therefore tempting to speculate that the CD154/CD40 dyad exerts a specific proinflammatory role at very early stages of tubular injury and that one of the underlying mechanisms involves the induction of IL-6 production.

The amplification of *XBP-1* mRNA splicing by CD154 in hypoxia suggests a link between the UPR and CD40 signaling. Alternative *XBP-1* mRNA splicing is induced by CD40 triggering [32, 57, 58]. Our results confirm that CD154 is an UPR regulator. In fact, many recent evidences point to the regulated nature of the UPR [3, 4]. The IRE-1/XBP-1 pathway is linked to inflammation, and activation of the IRE-1 pathway in kidney epithelial cells activates the NF-*κ*B pathway and secretion of inflammatory cytokines [38]. The regulation of XBP-1 splicing by CD40 signaling may therefore represent another regulatory interface in inflammation, which will deserve further studies.

As the CD40 pathway holds a central position in inflammation and contributes to multiple inflammatory disorders, inhibition of the CD40-CD154 pathway is an actively pursued strategy to treat inflammatory disorders. The administration of anti-CD154 neutralizing antibodies is effective in animal models but unfortunately causes thromboembolism, and various alternative strategies are sought for, such as the inhibition of CD40 signaling intermediates with peptides. We tested chloroquine as it was found to have anti-inflammatory properties, although the mechanisms involved remain ill-understood. Moreover, chloroquine confers protection in endotoxin shock and sepsis-induced AKI [59–62]. There is a strong association between the levels of systemic inflammatory mediators including IL-6 and the development of sepsis-induced AKI, suggesting a key responsibility of these mediators, including proinflammatory cytokines. IL-6 is a key contributor to AKI [9, 63]. Chloroquine can reduce the expression of IL-6 mRNA as in LPS-stimulated monocyte/macrophages; mechanisms were found to be partly due to accelerated mRNA decay [64]. The downregulatory action of chloroquine on the expression of inflammatory mediators can involve various mechanisms, such as at the mRNA level as shown, for example, for TNF-*α* [22, 65] or at the protein level [64, 66]. How chloroquine intersects with and inhibits the CD40 signaling remains to be further studied. However, the inhibitory effect of chloroquine on inflammatory cytokine production such as IL-6, coupled to the fact that chloroquine can also inhibit CD154 expression, as in T cells [67], suggests that this drug may have an interest as a useful adjunct in the clinical setting, for example, in conditions associated with AKI, such as in sepsis.

## Data Availability

The datasets generated for this study are available on request to the corresponding author.

## Supplementary Materials

Supplementary Materials Supplemental Material and Methods: primary antibodies used in this study: antibodies to BiP (Cell Signaling, Danvers, USA), calnexin (Stressgen, Ann Arbor, USA), eIF2*α* (Cell Signaling), phospho-eIF2*α* (Cell Signaling), ERK1/2 (Cell Signaling), phospho-ERK1/2 (Cell Signaling), JNK (Santa Cruz Biotechnology, Heidelberg, FRG), phospho-JNK (Santa Cruz Biotechnology), Akt (Cell Signaling), phospho-Akt (Cell Signaling), HIF-1*α* (Sigma-Aldrich, Saint-Quentin-Fallavier, France), *β*-tubulin (Abcam, Paris, France), and HIF-2*α* rabbit antibody D9E3 (Cell Signaling Technologies, London, UK) were used in Western blot experiments. Antibodies to human CD40 (H-10, Santa Cruz Biotechnology or mAb89 (a gift from Dr. Jacques Banchereau, Baylor Institute for Immunology Research, Dallas)) were used in flow cytometry experiments. Rabbit polyclonal anti-CD40 antibody (Biosource International, USA) was used in immunofluorescence. Supplemental Figure 1: CD154 amplifies XBP-1 mRNA splicing in anoxia-reoxygenation conditions. HK-2 cells were treated for 1 hour with 50 ng/mL antimycin A, a mitochondrial respiratory chain blocking agent, in the presence or not of rsCD154 at a concentration of 100 ng/mL; in these conditions, XBP-1 mRNA splicing was moderately induced but no effect of CD154 on XBP-1 mRNA splicing was detectable (R0). Antimycin A was removed and culture continued in normoxic conditions in the presence or not of rsCD154 and the spliced/unspliced ratio of XBP-1 (XBP1 s/u) mRNA in HK-2 cells measured at the indicated times by RT-qPCR (*n* = 4, ^∗^significant relatively to T0 condition, *p* < 0.05). Supplemental Figure 2: CD154 induces IL-6 mRNA expression in HK-2 cells in hypoxic conditions. HK-2 cells were grown under hypoxic conditions for 3 hours in Lumox tissue culture plates (A) or in standard tissue culture plates (B) in the presence or not of rsCD154; IL-6 protein was measured by ELISA in cell culture supernatants (A, *n* = 5, ^∗^significant relatively to control conditions, *p* < 0.05); B, *n* = 3, ^∗^significant relatively to control conditions, *p* < 0.05).
