# Supplementary material for: CD154 Induces Interleukin-6 Secretion by Kidney Tubular Epithelial Cells under Hypoxic Conditions: Inhibition by Chloroquine
Source: Mediators Inflamm. 2020 Jan 31;2020:6357046. doi: 10.1155/2020/6357046 (PMC7013356; doi:10.1155/2020/6357046)
Supplement: Supplementary Materials — Supplemental Material and Methods: primary antibodies used in this study: antibodies to BiP (Cell Signaling, Danvers, USA), calnexin (Stressgen, Ann Arbor, USA), eIF2α (Cell Signaling), phospho-eIF2α (Cell Signaling), ERK1/2 (Cell Signaling), phospho-ERK1/2 (Cell Signaling), JNK (Santa Cruz Biotechnology, Heidelberg, FRG), phospho-JNK (Santa Cruz Biotechnology), Akt (Cell Signaling), phospho-Akt (Cell Signaling), HIF-1α (Sigma-Aldrich, Saint-Quentin-Fallavier, France), β-tubulin (Abcam, Paris, France), and HIF-2α rabbit antibody D9E3 (Cell Signaling Technologies, London, UK) were used in Western blot experiments. Antibodies to human CD40 (H-10, Santa Cruz Biotechnology or mAb89 (a gift from Dr. Jacques Banchereau, Baylor Institute for Immunology Research, Dallas)) were used in flow cytometry experiments. Rabbit polyclonal anti-CD40 antibody (Biosource International, USA) was used in immunofluorescence. Supplemental Figure 1: CD154 amplifies XBP-1 mRNA splicing in anoxia-reoxygenation conditions. HK-2 cells were treated for 1 hour with 50 ng/mL antimycin A, a mitochondrial respiratory chain blocking agent, in the presence or not of rsCD154 at a concentration of 100 ng/mL; in these conditions, XBP-1 mRNA splicing was moderately induced but no effect of CD154 on XBP-1 mRNA splicing was detectable (R0). Antimycin A was removed and culture continued in normoxic conditions in the presence or not of rsCD154 and the spliced/unspliced ratio of XBP-1 (XBP1 s/u) mRNA in HK-2 cells measured at the indicated times by RT-qPCR (n = 4, ∗significant relatively to T0 condition, p < 0.05). Supplemental Figure 2: CD154 induces IL-6 mRNA expression in HK-2 cells in hypoxic conditions. HK-2 cells were grown under hypoxic conditions for 3 hours in Lumox tissue culture plates (A) or in standard tissue culture plates (B) in the presence or not of rsCD154; IL-6 protein was measured by ELISA in cell culture supernatants (A, n = 5, ∗significant relatively to control conditions, p < 0.05); B, n [file 6357046.f1.docx]

**Supplemental material and methods**

Primary antibodies used in this study

Antibodies to BiP (Cell Signalling ,Danvers, USA) calnexin (Stressgen, Ann Harbor, USA), eIF2α (Cell Signalling), phospho-eIF2α (Cell Signalling), ERK1/2 (Cell Signalling), phospho-ERK1/2 (Cell Signalling), JNK (Santa Cruz Biotechnology, Heidelberg, FRG), phospho-JNK (Santa Cruz Biotechnology), Akt (Cell Signalling), Phospho-Akt (Cell Signalling), HIF-1α (Sigma-Aldrich, Saint Quentin Fallaviers, France), β-tubulin (AbCam, Paris, France), HIF-2α rabbit antibody D9E3 (Cell Signaling Technologies, London, UK), were used in Western blot experiments. Antibodies to human CD40 (H-10, Santa-Cruz Biotechnology or or mAb89 (a gift from Dr Jacques Banchereau, Baylor Institute for Immunology Research, Dallas)) were used in flow cytometry experiments. Rabbit polyclonal anti-CD40 antibody (Biosource International, USA) was used in immunofluorescence.

**Supplemental Figure 1**

CD154 amplifies *XBP-1* mRNA splicing in anoxia-reoxygenation conditions.

HK-2 cells were treated for 1 hour with 50 ng/ml antimycin A, a mitochondrial respiratory chain blocking agent, in the presence or not of rsCD154 at a concentration of 100 ng/mL; in these conditions, *XBP-1* mRNA splicing was moderately induced but no effect of CD154 on *XBP-1* mRNA splicing was detectable (R0). Antimycin A was removed and culture continued in normoxic conditions in the presence or not of rsCD154 and the spliced/unspliced ratio of *XBP-1* (XBP1 s/u) mRNA in HK-2 cells measured at the indicated times by RT-qPCR (n=4, (*) significant relatively to T0 condition, p<0.05).

**Supplemental Figure 2**

CD154 induces IL-6 mRNA expression in HK-2 cells in hypoxic conditions.

HK-2 cells were grown under hypoxic conditions for 3 hours in Lumox tissue culture plates (A) or in standard tissue culture plates (B) in the presence or not of rsCD154; IL-6 protein was measured by ELISA in cell culture supernatants (A, n=5 (*) significant relatively to control conditions, p<0.05); B, n=3 (*) significant relatively to control conditions, p<0.05).
